# *N*-(4-Chloro­phen­yl)-4-(pyrimidin-2-yl)piperazine-1-carboxamide

**DOI:** 10.1107/S160053681103515X

**Published:** 2011-09-14

**Authors:** Yu-Feng Li

**Affiliations:** a Microscale Science Institute, Department of Chemistry and Chemical Engineering, Weifang University, Weifang 261061, People’s Republic of China

## Abstract

The title compound, C_15_H_16_ClN_5_O, contains two mol­ecules, *A* and *B*, in the asymmetric unit, in which the dihedral angles between the terminal aromatic rings are 42.41 (17) and 45.77 (18)°. The central six-membered ring in both mol­ecules has a chair conformation with equatorial substituents. In the crystal, mol­ecules are linked into [100] *C*(4) chains of alternating *A* and *B* mol­ecules by N—H⋯O hydrogen bonds.

## Related literature

For related structures, see: Li (2011*a* ▶,*b* ▶).
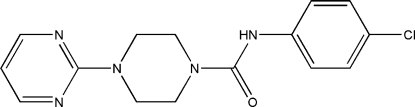


## Experimental

### 

#### Crystal data

C_15_H_16_ClN_5_O
*M* *_r_* = 317.78
Monoclinic, 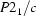

*a* = 9.992 (2) Å
*b* = 9.978 (2) Å
*c* = 31.197 (6) Å
β = 92.50 (3)°
*V* = 3107.3 (11) Å^3^
*Z* = 8
Mo *K*α radiation
μ = 0.26 mm^−1^
*T* = 293 K
0.22 × 0.21 × 0.19 mm

#### Data collection

Bruker SMART CCD diffractometer
29790 measured reflections
7075 independent reflections
3210 reflections with *I* > 2σ(*I*)
*R*_int_ = 0.070

#### Refinement

*R*[*F* ^2^ > 2σ(*F* ^2^)] = 0.058
*wR*(*F* ^2^) = 0.223
*S* = 0.93
7075 reflections
398 parameters
1 restraint
H-atom parameters constrained
Δρ_max_ = 0.31 e Å^−3^
Δρ_min_ = −0.35 e Å^−3^

### 

Data collection: *SMART* (Bruker, 1997 ▶); cell refinement: *SAINT* (Bruker, 1997 ▶); data reduction: *SAINT*; program(s) used to solve structure: *SHELXS97* (Sheldrick, 2008 ▶); program(s) used to refine structure: *SHELXL97* (Sheldrick, 2008 ▶); molecular graphics: *SHELXTL* (Sheldrick, 2008 ▶); software used to prepare material for publication: *SHELXTL*.

## Supplementary Material

Crystal structure: contains datablock(s) global, I. DOI: 10.1107/S160053681103515X/hb6389sup1.cif

Structure factors: contains datablock(s) I. DOI: 10.1107/S160053681103515X/hb6389Isup2.hkl

Supplementary material file. DOI: 10.1107/S160053681103515X/hb6389Isup3.cml

Additional supplementary materials:  crystallographic information; 3D view; checkCIF report

## Figures and Tables

**Table 1:** Hydrogen-bond geometry (Å, °)

*D*—H⋯*A*	*D*—H	H⋯*A*	*D*⋯*A*	*D*—H⋯*A*
N3*B*—H3*BA*⋯O1*A*	0.86	2.18	3.030 (3)	172
N3*A*—H3*AA*⋯O1*B*^i^	0.86	2.26	3.090 (3)	163

Symmetry code: (i) 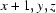.

**Fig. 1.**
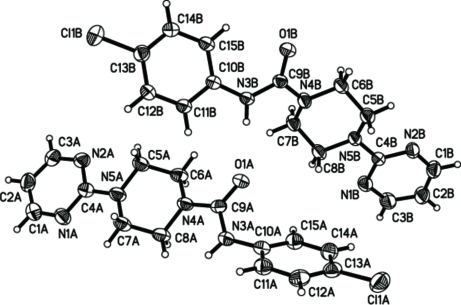
The structure of the title compound showing 30% probability displacement ellipsoids.

## supplementary crystallographic information

### Comment

The crystal structure of the title compound is presented herein. The molecular structure of the title compound is shown in Fig. 1. The six-membered rings (N4A, N5A, C5A, C6A, C7A, C8A) (N4B, N5B, C5B, C6B, C7B, C8B) are in chair conformations. The structures of related compound have already been determined Li (2011*a*,*b*).

### Experimental

A mixture of 2-(piperazin-1-yl)pyrimidine (0.05 mol), and (4-chlorophenyl)carbamic chloride (0.05 mol) was stirred in refluxing ethanol (15 ml) for 6 h to afford the title compound (0.04 mol, yield 80%). Colourless blocks of the title compound were obtained by recrystallization from ethanol at room temperature.

### Refinement

H atoms were fixed geometrically and allowed to ride on their attached atoms, with C—H distances = 0.93–0.97 Å; N—H = 0.86Å and with *U*_iso_(H) = 1.2*U*_eq_(C,*N*) or 1.5*U*_eq_(C_methyl_).

### Crystal data

**Table:**

C_15_H_16_ClN_5_O	*F*(000) = 1328
*M**_r_* = 317.78	*D*_x_ = 1.359 Mg m^−^^3^
Monoclinic, *P*2_1_/*c*	Mo *K*α radiation, λ = 0.71073 Å
Hall symbol: -P 2ybc	Cell parameters from 3210 reflections
*a* = 9.992 (2) Å	θ = 3.1–26.9°
*b* = 9.978 (2) Å	µ = 0.26 mm^−^^1^
*c* = 31.197 (6) Å	*T* = 293 K
β = 92.50 (3)°	Block, colorless
*V* = 3107.3 (11) Å^3^	0.22 × 0.21 × 0.19 mm
*Z* = 8	

### Data collection

**Table:**

Bruker SMART CCD diffractometer	3210 reflections with *I* > 2σ(*I*)
Radiation source: fine-focus sealed tube	*R*_int_ = 0.070
graphite	θ_max_ = 27.5°, θ_min_ = 3.1°
φ and ω scans	*h* = −12→12
29790 measured reflections	*k* = −12→12
7075 independent reflections	*l* = −40→40

### Refinement

**Table:**

Refinement on *F*^2^	Secondary atom site location: difference Fourier map
Least-squares matrix: full	Hydrogen site location: inferred from neighbouring sites
*R*[*F*^2^ > 2σ(*F*^2^)] = 0.058	H-atom parameters constrained
*wR*(*F*^2^) = 0.223	*w* = 1/[σ^2^(*F*_o_^2^) + (0.1316*P*)^2^] where *P* = (*F*_o_^2^ + 2*F*_c_^2^)/3
*S* = 0.93	(Δ/σ)_max_ < 0.001
7075 reflections	Δρ_max_ = 0.31 e Å^−^^3^
398 parameters	Δρ_min_ = −0.35 e Å^−^^3^
1 restraint	Extinction correction: *SHELXL97* (Sheldrick, 2008), Fc^*^=kFc[1+0.001xFc^2^λ^3^/sin(2θ)]^-1/4^
Primary atom site location: structure-invariant direct methods	Extinction coefficient: 0.0100 (14)

### Special details

**Table:**

Geometry. All e.s.d.'s (except the e.s.d. in the dihedral angle between two l.s. planes) are estimated using the full covariance matrix. The cell e.s.d.'s are taken into account individually in the estimation of e.s.d.'s in distances, angles and torsion angles; correlations between e.s.d.'s in cell parameters are only used when they are defined by crystal symmetry. An approximate (isotropic) treatment of cell e.s.d.'s is used for estimating e.s.d.'s involving l.s. planes.
Refinement. Refinement of *F*^2^ against ALL reflections. The weighted *R*-factor *wR* and goodness of fit *S* are based on *F*^2^, conventional *R*-factors *R* are based on *F*, with *F* set to zero for negative *F*^2^. The threshold expression of *F*^2^ > σ(*F*^2^) is used only for calculating *R*-factors(gt) *etc*. and is not relevant to the choice of reflections for refinement. *R*-factors based on *F*^2^ are statistically about twice as large as those based on *F*, and *R*- factors based on ALL data will be even larger.

### Fractional atomic coordinates and isotropic or equivalent isotropic displacement parameters (Å^2^)

**Table:**

	*x*	*y*	*z*	*U*_iso_*/*U*_eq_	
Cl1A	0.63361 (12)	0.48186 (12)	0.04864 (3)	0.0889 (4)	
Cl1B	0.29934 (11)	0.41025 (14)	0.44307 (3)	0.0920 (4)	
O1B	0.02837 (19)	0.2901 (2)	0.24124 (7)	0.0555 (6)	
O1A	0.51755 (19)	0.1666 (3)	0.23043 (7)	0.0644 (7)	
N3B	0.2439 (2)	0.2371 (3)	0.26209 (8)	0.0507 (7)	
H3BA	0.3181	0.2098	0.2521	0.061*	
N5B	0.1716 (2)	−0.0057 (3)	0.12470 (8)	0.0521 (7)	
C10B	0.2507 (3)	0.2866 (3)	0.30407 (9)	0.0454 (7)	
N4A	0.6814 (2)	0.1119 (3)	0.27995 (9)	0.0552 (7)	
C9A	0.6367 (3)	0.1683 (3)	0.24220 (10)	0.0507 (8)	
N3A	0.7298 (2)	0.2315 (3)	0.21896 (8)	0.0560 (7)	
H3AA	0.8101	0.2368	0.2299	0.067*	
C9B	0.1331 (3)	0.2269 (3)	0.23506 (9)	0.0470 (7)	
N2B	0.1097 (3)	−0.0434 (3)	0.05334 (8)	0.0589 (7)	
N4B	0.1467 (2)	0.1485 (3)	0.20004 (8)	0.0527 (7)	
N5A	0.7610 (3)	0.0069 (3)	0.36110 (9)	0.0636 (8)	
C15B	0.1393 (3)	0.3037 (3)	0.32880 (10)	0.0557 (8)	
H15A	0.0539	0.2894	0.3167	0.067*	
C4B	0.1935 (3)	−0.0696 (3)	0.08677 (10)	0.0500 (8)	
N2A	0.7043 (3)	−0.1024 (3)	0.42290 (9)	0.0676 (8)	
C14B	0.1551 (3)	0.3419 (4)	0.37141 (11)	0.0608 (9)	
H14A	0.0802	0.3530	0.3877	0.073*	
C6A	0.5937 (3)	0.0212 (4)	0.30213 (11)	0.0606 (9)	
H6AA	0.6087	−0.0700	0.2927	0.073*	
H6AB	0.5010	0.0441	0.2951	0.073*	
C12B	0.3911 (3)	0.3483 (4)	0.36561 (10)	0.0590 (9)	
H12A	0.4761	0.3635	0.3780	0.071*	
C15A	0.6295 (3)	0.2246 (4)	0.14602 (11)	0.0588 (8)	
H15B	0.5926	0.1411	0.1515	0.071*	
N1B	0.2941 (3)	−0.1586 (3)	0.08730 (9)	0.0700 (9)	
C8B	0.2786 (3)	−0.0038 (4)	0.15814 (11)	0.0631 (9)	
H8BA	0.3438	0.0642	0.1514	0.076*	
H8BB	0.3238	−0.0899	0.1589	0.076*	
C11A	0.7593 (3)	0.4140 (4)	0.16934 (11)	0.0602 (9)	
H11A	0.8095	0.4587	0.1907	0.072*	
C10A	0.7036 (3)	0.2897 (3)	0.17782 (10)	0.0517 (8)	
N1A	0.9289 (3)	−0.0319 (3)	0.41271 (10)	0.0712 (9)	
C4A	0.7992 (3)	−0.0428 (3)	0.40047 (11)	0.0575 (8)	
C6B	0.0374 (3)	0.1454 (4)	0.16727 (10)	0.0597 (9)	
H6BA	−0.0082	0.2312	0.1665	0.072*	
H6BB	−0.0269	0.0771	0.1745	0.072*	
C13B	0.2796 (3)	0.3633 (4)	0.38956 (10)	0.0581 (8)	
C11B	0.3761 (3)	0.3107 (3)	0.32327 (10)	0.0542 (8)	
H11B	0.4516	0.3013	0.3072	0.065*	
C14A	0.6097 (3)	0.2824 (4)	0.10616 (11)	0.0639 (9)	
H14B	0.5594	0.2384	0.0847	0.077*	
C12A	0.7404 (4)	0.4714 (4)	0.12927 (13)	0.0668 (10)	
H12B	0.7786	0.5540	0.1234	0.080*	
C8A	0.8233 (3)	0.0853 (4)	0.29136 (12)	0.0640 (10)	
H8AA	0.8788	0.1497	0.2771	0.077*	
H8AB	0.8469	−0.0036	0.2816	0.077*	
C13A	0.6650 (3)	0.4057 (4)	0.09834 (11)	0.0612 (9)	
C5A	0.6198 (3)	0.0302 (4)	0.34979 (11)	0.0652 (10)	
H5AA	0.5941	0.1182	0.3597	0.078*	
H5AB	0.5658	−0.0359	0.3639	0.078*	
C7B	0.2247 (3)	0.0249 (4)	0.20118 (10)	0.0588 (9)	
H7BA	0.1684	−0.0490	0.2096	0.071*	
H7BB	0.2983	0.0331	0.2223	0.071*	
C7A	0.8498 (3)	0.0948 (4)	0.33886 (12)	0.0634 (9)	
H7AA	0.9420	0.0703	0.3459	0.076*	
H7AB	0.8366	0.1865	0.3481	0.076*	
C5B	0.0904 (3)	0.1158 (4)	0.12395 (10)	0.0599 (9)	
H5BA	0.0159	0.1053	0.1032	0.072*	
H5BB	0.1442	0.1907	0.1149	0.072*	
C2B	0.2315 (4)	−0.2037 (5)	0.01431 (12)	0.0776 (12)	
H2BB	0.2456	−0.2495	−0.0111	0.093*	
C3A	0.7452 (4)	−0.1557 (4)	0.46014 (12)	0.0749 (11)	
H3AB	0.6819	−0.1974	0.4766	0.090*	
C3B	0.3093 (4)	−0.2242 (4)	0.05043 (12)	0.0782 (12)	
H3BB	0.3773	−0.2877	0.0495	0.094*	
C1B	0.1308 (4)	−0.1113 (4)	0.01751 (11)	0.0706 (10)	
H1BB	0.0746	−0.0952	−0.0065	0.085*	
C2A	0.8744 (4)	−0.1528 (5)	0.47566 (12)	0.0827 (12)	
H2AB	0.9009	−0.1918	0.5018	0.099*	
C1A	0.9632 (4)	−0.0888 (5)	0.45040 (13)	0.0818 (12)	
H1AB	1.0525	−0.0847	0.4600	0.098*	

### Atomic displacement parameters (Å^2^)

**Table:**

	*U*^11^	*U*^22^	*U*^33^	*U*^12^	*U*^13^	*U*^23^
Cl1A	0.0949 (8)	0.1019 (9)	0.0713 (6)	0.0216 (6)	0.0196 (6)	0.0231 (6)
Cl1B	0.0939 (8)	0.1238 (10)	0.0581 (6)	0.0002 (7)	0.0017 (5)	−0.0239 (6)
O1B	0.0418 (11)	0.0642 (15)	0.0604 (13)	0.0035 (10)	0.0026 (10)	−0.0023 (11)
O1A	0.0386 (11)	0.0899 (19)	0.0650 (14)	−0.0018 (11)	0.0045 (10)	0.0005 (12)
N3B	0.0339 (11)	0.0680 (19)	0.0502 (14)	−0.0014 (12)	0.0025 (11)	−0.0088 (13)
N5B	0.0467 (13)	0.0621 (18)	0.0471 (14)	0.0123 (12)	−0.0037 (11)	−0.0020 (12)
C10B	0.0389 (14)	0.0457 (18)	0.0517 (17)	−0.0001 (12)	0.0028 (13)	0.0005 (13)
N4A	0.0373 (12)	0.0663 (19)	0.0624 (17)	−0.0059 (12)	0.0064 (12)	0.0069 (14)
C9A	0.0434 (16)	0.055 (2)	0.0547 (18)	0.0007 (14)	0.0103 (14)	−0.0036 (14)
N3A	0.0396 (12)	0.070 (2)	0.0583 (16)	−0.0062 (12)	0.0037 (12)	0.0046 (14)
C9B	0.0385 (14)	0.052 (2)	0.0503 (16)	−0.0029 (13)	0.0045 (13)	0.0051 (14)
N2B	0.0588 (15)	0.068 (2)	0.0490 (15)	0.0095 (14)	−0.0062 (13)	−0.0030 (13)
N4B	0.0517 (14)	0.0561 (18)	0.0495 (14)	0.0120 (12)	−0.0087 (12)	−0.0054 (12)
N5A	0.0435 (14)	0.078 (2)	0.0695 (19)	−0.0052 (13)	0.0042 (13)	0.0220 (15)
C15B	0.0386 (14)	0.068 (2)	0.060 (2)	0.0003 (14)	0.0005 (14)	−0.0053 (16)
C4B	0.0419 (15)	0.060 (2)	0.0479 (17)	0.0014 (14)	−0.0001 (13)	−0.0021 (14)
N2A	0.0609 (17)	0.079 (2)	0.0631 (18)	0.0012 (15)	0.0086 (15)	0.0121 (16)
C14B	0.0476 (17)	0.076 (3)	0.059 (2)	0.0061 (16)	0.0079 (15)	−0.0070 (17)
C6A	0.0465 (16)	0.062 (2)	0.074 (2)	−0.0094 (15)	0.0109 (16)	0.0070 (17)
C12B	0.0430 (15)	0.076 (3)	0.0575 (19)	−0.0034 (16)	−0.0033 (15)	−0.0061 (17)
C15A	0.0574 (18)	0.052 (2)	0.067 (2)	−0.0098 (16)	0.0040 (17)	−0.0018 (16)
N1B	0.0608 (16)	0.088 (2)	0.0605 (17)	0.0213 (16)	−0.0051 (14)	−0.0158 (16)
C8B	0.0472 (17)	0.080 (3)	0.061 (2)	0.0148 (16)	−0.0105 (15)	−0.0096 (17)
C11A	0.0569 (18)	0.056 (2)	0.068 (2)	−0.0057 (16)	0.0055 (17)	−0.0028 (17)
C10A	0.0410 (14)	0.054 (2)	0.0603 (19)	0.0014 (14)	0.0073 (14)	−0.0001 (15)
N1A	0.0554 (17)	0.084 (2)	0.073 (2)	−0.0001 (15)	−0.0067 (15)	0.0107 (17)
C4A	0.0529 (18)	0.056 (2)	0.064 (2)	0.0016 (16)	0.0052 (16)	0.0032 (16)
C6B	0.0459 (16)	0.074 (3)	0.0585 (19)	0.0134 (16)	−0.0086 (15)	−0.0088 (17)
C13B	0.0606 (19)	0.058 (2)	0.0557 (19)	0.0054 (16)	0.0009 (16)	−0.0051 (15)
C11B	0.0397 (15)	0.069 (2)	0.0540 (18)	−0.0037 (14)	0.0040 (14)	−0.0083 (15)
C14A	0.0631 (19)	0.067 (3)	0.061 (2)	−0.0013 (18)	0.0019 (17)	−0.0066 (17)
C12A	0.066 (2)	0.058 (2)	0.078 (2)	−0.0060 (18)	0.017 (2)	0.0043 (19)
C8A	0.0411 (16)	0.072 (3)	0.080 (2)	−0.0002 (16)	0.0097 (17)	0.0142 (19)
C13A	0.0574 (18)	0.063 (2)	0.064 (2)	0.0114 (17)	0.0162 (17)	0.0040 (17)
C5A	0.0439 (16)	0.082 (3)	0.070 (2)	−0.0039 (17)	0.0027 (16)	0.0152 (19)
C7B	0.0584 (18)	0.059 (2)	0.0578 (19)	0.0166 (16)	−0.0126 (15)	−0.0052 (16)
C7A	0.0417 (16)	0.069 (2)	0.080 (2)	−0.0091 (16)	0.0011 (16)	0.0175 (19)
C5B	0.0575 (18)	0.066 (2)	0.0557 (19)	0.0161 (17)	−0.0046 (15)	−0.0018 (16)
C2B	0.082 (2)	0.093 (3)	0.057 (2)	0.017 (2)	−0.004 (2)	−0.018 (2)
C3A	0.080 (2)	0.090 (3)	0.055 (2)	0.002 (2)	0.0078 (19)	0.0073 (19)
C3B	0.071 (2)	0.091 (3)	0.072 (2)	0.026 (2)	−0.002 (2)	−0.022 (2)
C1B	0.078 (2)	0.079 (3)	0.0531 (19)	0.009 (2)	−0.0061 (18)	−0.0083 (18)
C2A	0.093 (3)	0.099 (4)	0.055 (2)	0.012 (3)	−0.008 (2)	0.006 (2)
C1A	0.070 (2)	0.104 (4)	0.070 (2)	0.005 (2)	−0.009 (2)	0.004 (2)

### Geometric parameters (Å, °)

**Table:**

Cl1A—C13A	1.743 (4)	C15A—C14A	1.377 (5)
Cl1B—C13B	1.737 (3)	C15A—H15B	0.9300
O1B—C9B	1.244 (3)	N1B—C3B	1.338 (4)
O1A—C9A	1.230 (4)	C8B—C7B	1.496 (5)
N3B—C9B	1.366 (4)	C8B—H8BA	0.9700
N3B—C10B	1.399 (4)	C8B—H8BB	0.9700
N3B—H3BA	0.8600	C11A—C12A	1.380 (5)
N5B—C4B	1.370 (4)	C11A—C10A	1.390 (5)
N5B—C5B	1.458 (4)	C11A—H11A	0.9300
N5B—C8B	1.461 (4)	N1A—C1A	1.337 (5)
C10B—C11B	1.385 (4)	N1A—C4A	1.340 (4)
C10B—C15B	1.392 (4)	C6B—C5B	1.502 (4)
N4A—C9A	1.363 (4)	C6B—H6BA	0.9700
N4A—C6A	1.456 (4)	C6B—H6BB	0.9700
N4A—C8A	1.471 (4)	C11B—H11B	0.9300
C9A—N3A	1.360 (4)	C14A—C13A	1.375 (5)
N3A—C10A	1.422 (4)	C14A—H14B	0.9300
N3A—H3AA	0.8600	C12A—C13A	1.365 (5)
C9B—N4B	1.356 (4)	C12A—H12B	0.9300
N2B—C1B	1.332 (4)	C8A—C7A	1.497 (5)
N2B—C4B	1.334 (4)	C8A—H8AA	0.9700
N4B—C7B	1.458 (4)	C8A—H8AB	0.9700
N4B—C6B	1.463 (4)	C5A—H5AA	0.9700
N5A—C4A	1.364 (4)	C5A—H5AB	0.9700
N5A—C7A	1.446 (4)	C7B—H7BA	0.9700
N5A—C5A	1.458 (4)	C7B—H7BB	0.9700
C15B—C14B	1.385 (4)	C7A—H7AA	0.9700
C15B—H15A	0.9300	C7A—H7AB	0.9700
C4B—N1B	1.341 (4)	C5B—H5BA	0.9700
N2A—C3A	1.326 (5)	C5B—H5BB	0.9700
N2A—C4A	1.342 (4)	C2B—C3B	1.356 (5)
C14B—C13B	1.360 (5)	C2B—C1B	1.372 (5)
C14B—H14A	0.9300	C2B—H2BB	0.9300
C6A—C5A	1.501 (5)	C3A—C2A	1.359 (5)
C6A—H6AA	0.9700	C3A—H3AB	0.9300
C6A—H6AB	0.9700	C3B—H3BB	0.9300
C12B—C11B	1.375 (4)	C1B—H1BB	0.9300
C12B—C13B	1.377 (4)	C2A—C1A	1.370 (6)
C12B—H12A	0.9300	C2A—H2AB	0.9300
C15A—C10A	1.375 (5)	C1A—H1AB	0.9300
			
C9B—N3B—C10B	127.5 (2)	N4B—C6B—H6BA	109.5
C9B—N3B—H3BA	116.2	C5B—C6B—H6BA	109.5
C10B—N3B—H3BA	116.2	N4B—C6B—H6BB	109.5
C4B—N5B—C5B	118.9 (3)	C5B—C6B—H6BB	109.5
C4B—N5B—C8B	118.9 (2)	H6BA—C6B—H6BB	108.1
C5B—N5B—C8B	113.0 (3)	C14B—C13B—C12B	120.3 (3)
C11B—C10B—C15B	117.9 (3)	C14B—C13B—Cl1B	120.4 (2)
C11B—C10B—N3B	118.2 (2)	C12B—C13B—Cl1B	119.3 (3)
C15B—C10B—N3B	123.7 (3)	C12B—C11B—C10B	121.4 (3)
C9A—N4A—C6A	119.0 (3)	C12B—C11B—H11B	119.3
C9A—N4A—C8A	124.1 (2)	C10B—C11B—H11B	119.3
C6A—N4A—C8A	111.5 (3)	C13A—C14A—C15A	119.5 (4)
O1A—C9A—N3A	121.5 (3)	C13A—C14A—H14B	120.3
O1A—C9A—N4A	122.0 (3)	C15A—C14A—H14B	120.3
N3A—C9A—N4A	116.5 (3)	C13A—C12A—C11A	119.4 (4)
C9A—N3A—C10A	124.6 (3)	C13A—C12A—H12B	120.3
C9A—N3A—H3AA	117.7	C11A—C12A—H12B	120.3
C10A—N3A—H3AA	117.7	N4A—C8A—C7A	110.8 (3)
O1B—C9B—N4B	122.2 (3)	N4A—C8A—H8AA	109.5
O1B—C9B—N3B	122.1 (3)	C7A—C8A—H8AA	109.5
N4B—C9B—N3B	115.7 (3)	N4A—C8A—H8AB	109.5
C1B—N2B—C4B	116.1 (3)	C7A—C8A—H8AB	109.5
C9B—N4B—C7B	122.7 (3)	H8AA—C8A—H8AB	108.1
C9B—N4B—C6B	118.5 (2)	C12A—C13A—C14A	121.2 (4)
C7B—N4B—C6B	112.4 (3)	C12A—C13A—Cl1A	119.5 (3)
C4A—N5A—C7A	119.9 (3)	C14A—C13A—Cl1A	119.3 (3)
C4A—N5A—C5A	120.4 (3)	N5A—C5A—C6A	110.8 (3)
C7A—N5A—C5A	113.3 (3)	N5A—C5A—H5AA	109.5
C14B—C15B—C10B	120.3 (3)	C6A—C5A—H5AA	109.5
C14B—C15B—H15A	119.8	N5A—C5A—H5AB	109.5
C10B—C15B—H15A	119.8	C6A—C5A—H5AB	109.5
N2B—C4B—N1B	125.7 (3)	H5AA—C5A—H5AB	108.1
N2B—C4B—N5B	117.7 (3)	N4B—C7B—C8B	110.7 (3)
N1B—C4B—N5B	116.5 (3)	N4B—C7B—H7BA	109.5
C3A—N2A—C4A	115.9 (3)	C8B—C7B—H7BA	109.5
C13B—C14B—C15B	120.4 (3)	N4B—C7B—H7BB	109.5
C13B—C14B—H14A	119.8	C8B—C7B—H7BB	109.5
C15B—C14B—H14A	119.8	H7BA—C7B—H7BB	108.1
N4A—C6A—C5A	110.6 (3)	N5A—C7A—C8A	110.5 (3)
N4A—C6A—H6AA	109.5	N5A—C7A—H7AA	109.5
C5A—C6A—H6AA	109.5	C8A—C7A—H7AA	109.5
N4A—C6A—H6AB	109.5	N5A—C7A—H7AB	109.5
C5A—C6A—H6AB	109.5	C8A—C7A—H7AB	109.5
H6AA—C6A—H6AB	108.1	H7AA—C7A—H7AB	108.1
C11B—C12B—C13B	119.6 (3)	N5B—C5B—C6B	111.5 (3)
C11B—C12B—H12A	120.2	N5B—C5B—H5BA	109.3
C13B—C12B—H12A	120.2	C6B—C5B—H5BA	109.3
C10A—C15A—C14A	120.3 (3)	N5B—C5B—H5BB	109.3
C10A—C15A—H15B	119.8	C6B—C5B—H5BB	109.3
C14A—C15A—H15B	119.8	H5BA—C5B—H5BB	108.0
C3B—N1B—C4B	115.3 (3)	C3B—C2B—C1B	115.9 (3)
N5B—C8B—C7B	111.2 (3)	C3B—C2B—H2BB	122.1
N5B—C8B—H8BA	109.4	C1B—C2B—H2BB	122.1
C7B—C8B—H8BA	109.4	N2A—C3A—C2A	123.7 (4)
N5B—C8B—H8BB	109.4	N2A—C3A—H3AB	118.1
C7B—C8B—H8BB	109.4	C2A—C3A—H3AB	118.1
H8BA—C8B—H8BB	108.0	N1B—C3B—C2B	123.9 (4)
C12A—C11A—C10A	120.2 (4)	N1B—C3B—H3BB	118.0
C12A—C11A—H11A	119.9	C2B—C3B—H3BB	118.0
C10A—C11A—H11A	119.9	N2B—C1B—C2B	123.1 (4)
C15A—C10A—C11A	119.5 (3)	N2B—C1B—H1BB	118.4
C15A—C10A—N3A	122.0 (3)	C2B—C1B—H1BB	118.4
C11A—C10A—N3A	118.5 (3)	C3A—C2A—C1A	115.7 (4)
C1A—N1A—C4A	115.1 (3)	C3A—C2A—H2AB	122.2
N1A—C4A—N2A	125.7 (3)	C1A—C2A—H2AB	122.2
N1A—C4A—N5A	117.2 (3)	N1A—C1A—C2A	123.8 (4)
N2A—C4A—N5A	117.0 (3)	N1A—C1A—H1AB	118.1
N4B—C6B—C5B	110.6 (3)	C2A—C1A—H1AB	118.1

### Hydrogen-bond geometry (Å, °)

**Table:**

*D*—H···*A*	*D*—H	H···*A*	*D*···*A*	*D*—H···*A*
N3B—H3BA···O1A	0.86	2.18	3.030 (3)	172
N3A—H3AA···O1B^i^	0.86	2.26	3.090 (3)	163

Symmetry codes: (i) *x*+1, *y*, *z*.
